# Study on the application of thermo-optical surface monitoring + X-ray guidance system in DIBH for breast cancer

**DOI:** 10.3389/fonc.2026.1880922

**Published:** 2026-07-07

**Authors:** Tian Tian, Jicheng Zhang, Fei Chen, Xiaoqin Gong, Wuyang Yang, Jian Huang, Tao You

**Affiliations:** Tumor Center, Affiliated Hospital of Jiangsu University, Zhenjiang, China

**Keywords:** breast cancer, deep inspiration breath-hold, radiotherapy, setup accuracy, thermo-optical surface monitoring + X-ray guidance system

## Abstract

**Objective:**

To evaluate the clinical value of thermo-optical surface monitoring + X-ray guidance system(ExacTrac Dynamic, ETD) in assessing inter-fraction setup accuracy and intra-fraction motion monitoring during deep inspiration breath-hold (DIBH) radiotherapy after breast-conserving surgery for breast cancer.

**Methods:**

Clinical data were collected from 31 female patients with breast cancer who had undergone breast−conserving surgery. Cone−beam computed tomography (CBCT) was used to verify inter−fraction setup during the first three treatment fractions, as well as the 6th, 9th, 12th, and 15th fractions. Triple thermo−optical surface tracking, intra−fraction CBCT, and X−ray imaging were employed to monitor intra−fraction motion. Setup errors and treatment duration were recorded. Linear mixed−effects models were applied to analyze inter−fraction setup errors derived from CBCT and intra−fraction errors measured by X−ray.

**Results:**

After X-ray correction, mean setup errors in all dimensions were not significantly different from zero (*P*>0.05) and were independent of breast volume. Treatment duration had no significant impact on intra-fraction deviations. Surface monitoring detected mid-treatment drift, which was corrected by X-ray guidance, keeping all values within clinical tolerance. The radiation dose from X-ray planar imaging was substantially lower than from CBCT. Calculated planning target volume (PTV) margins (van Herk formula) were 5.10 mm (lateral), 5.22 mm (longitudinal), and 5.78 mm (vertical).

**Conclusion:**

ETD provides setup accuracy comparable to CBCT with lower radiation dose, making it appropriate for inter−fraction verification. It allows stable real−time intra−fraction monitoring and can enhance treatment precision with mid−treatment X−ray correction.

## 1 Introduction

According to Global Cancer Statistics 2022, breast cancer ranks first in incidence and mortality among female malignancies globally, constituting a severe threat to women’s health and lives (1). Radiotherapy is one of the core treatments for breast cancer (2). However, common radiotherapy-related adverse events, including radiation-induced heart disease and radiation-induced lung injury (3, 4), which may inflict additional harm on patients. With the development of radiotherapy technologies, the Deep Inspiration Breath-Hold (DIBH) technique, which enlarges the distance between the heart, lungs and chest wall via respiratory control, has been proven to effectively reduce cardiac and pulmonary radiation exposure (5–7), thus significantly decreasing the risk of the aforementioned complications. To ensure the reliable implementation of DIBH, clinical practice commonly adopts respiratory motion management and monitoring systems, such as Real-time Position Management (RPM), Active Breathing Coordinator (ABC) and Optical Surface Monitoring System (OSMS) (8–11).

In recent years, surface guided radiation therapy (SGRT) has gradually been applied to the positioning and motion monitoring of patients with breast DIBH (12). AAPM TG-302 also regards breast DIBH radiotherapy as one of the important clinical applications of SGRT (13). But most SGRT systems mainly provide external surrogate monitoring. The thermal surface monitoring + X-ray guidance system (ExacTrac Dynamic, ETD) combines traditional optical surface monitoring with thermal imaging positioning and tracking, as well as three-dimensional X-ray imaging verification. It conducts real-time surface tracking, monitoring, and six-dimensional position correction during the patient’s treatment process (14, 15). However, at present, there is still a lack of sufficient clinical data regarding the relationship between ETD, cone-beam computed tomography (CBCT) verification results, mid-course treatment positional drift, and image dose in breast cancer DIBH radiotherapy. Therefore, it is necessary to conduct an assessment based on the actual process of our center.

This study applied the ETD to breast cancer DIBH radiotherapy, using CBCT as an independent imaging reference. Statistical analysis was conducted to evaluate the inter-fraction and intra-fraction setup deviations, treatment duration correlation, and image-verified dose. The study focused on assessing the feasibility of this system in low-dose image guidance, real-time surface monitoring, and mid-course re-correction during treatment, providing a reference for clinical process optimization.

## 2 Materials and methods

### 2.1 Clinical data

A total of 31 female patients with breast cancer who underwent breast-conserving surgery were enrolled in Affiliated Hospital of Jiangsu University between May and November 2025. Among these patients, 14 had left-sided breast cancer and 17 had right-sided breast cancer. Patient age ranged from 32.0 to 66.0 years, with a median age of 46.0 years. The affected breast volume was 349.0–2160.0 ml (median 811.0 ml), while the contralateral breast volume was 117.0–960.0 ml (median 349.0 ml). All patients had no history of severe cardiopulmonary disorders or ipsilateral chest radiotherapy. Their Karnofsky Performance Status (KPS) scores were ≥ 90, with normal bilateral upper arm mobility. All participants met the postural requirements of treatment and could cooperate with imaging examinations.

### 2.2 Instrumentation

**Figure 1:**
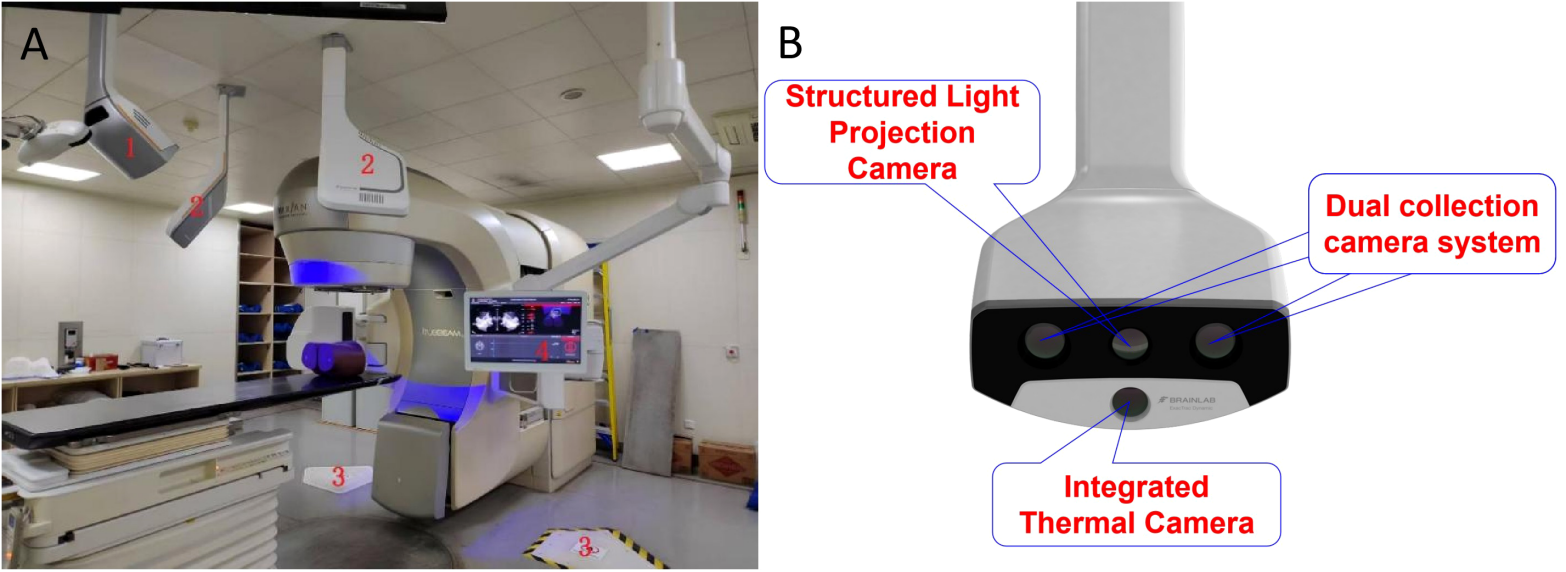
ETD integrated guidance system. **(A)** Schematic diagram of ETD:1. Thermal - optical surface imaging system, 2. X-ray detector, 3. X-ray generator, 4. Indoor control panel. The blue color represents the structured light projected by the optical surface system. **(B)** Schematic diagram of the thermal + optical surface imaging system.

A breast support bracket (Guangzhou Keleiruidi); a positioning CT scanner (Brilliance CT big bore, Philips); Thermo-optical Surface Monitoring + X-ray Guidance System ETD (Brian LAB, Germany) (**Figure 1A**); and a linear accelerator (Truebeam STx, Varian, USA).

Working principle of ETD: As a comprehensive image-guided system, it integrates X-ray imaging with multimodal imaging modalities including thermal and optical surface imaging. The thermo-optical surface imaging system employs a combined camera mounted above the distal end of the accelerator bed to simultaneously acquire the patient’s 3D optical surface contour and 3D thermal images (**Figure 1B**). Image fusion of these two modalities yields a high-accuracy thermo-optical surface model, enabling real-time dynamic monitoring. ETD automatically calculates the six-dimensional setup errors between the patient’s intra-treatment body surface and the surface structure reconstructed from positioning CT data. It monitors patient surface motion in real time and controls the accelerator beam output via predefined tolerance thresholds. The X-ray imaging system comprises two sets of X-ray generators and detectors, which can acquire X-ray images throughout treatment to verify six-dimensional setup errors, and subsequently trigger the accelerator to perform position calibration. ETD can operate either synchronously or independently. When used in combination, thermo-optical surface monitoring is performed on the basis of the patient surface calibrated by the X-ray imaging system, further enhancing monitoring precision. When used alone, thermo-optical surface imaging system is a non-radiation monitoring modality.

### 2.3 Methods

#### 2.3.1 Patient respiratory training

All patients exhibited normal pulmonary function and satisfactory communication skills and were able to comply with DIBH procedures. Before CT simulation positioning, repeated respiratory training was conducted. Patients were placed in the supine position with both arms raised above the head and instructed to perform thoracic breathing (nasal inhalation with closed lips). The breath-hold duration after deep inspiration exceeded 25 s; a stable breath-hold of over 40 s, repeatable at least 8 times, was deemed optimal. The vertical elevation of the affected chest wall between DIBH and free-breathing (FB) states was greater than 5 mm, with a target optimal value of ≥ 10 mm.

#### 2.3.2 CT Simulation positioning and image acquisition

All patients were immobilized using a dedicated breast support bracket. Patients were positioned supine on the bracket with their backs firmly secured against it. The bracket maintained both upper extremities in a comfortable, elevated position, while hip supports and foot supports were utilized to fix the patient’s hips and lower limbs. The reproducibility of respiratory training and breath-hold duration was reconfirmed under FB conditions. CT scans were acquired separately in both FB and DIBH states, with scanning parameters set as follows: 120 kV, 100 mAs, 3 mm slice thickness, and spiral scanning mode. Breath-hold stability was monitored throughout scanning, with a tolerance of ± 2 mm relative to the breath-hold baseline.

#### 2.3.3 Image fusion and plan design

FB images were registered and fused with DIBH positioning images. The volume of interest (VOI) was delineated as the T1–T12 spinal plane, extending anteriorly to half of the mediastinum, posteriorly to the carbon fiber plate, and encompassing bilateral mediastinal tissues. The FB outer contour was transferred to DIBH images, with the affected chest wall confirmed to have a vertical elevation > 5 mm compared with FB. Treatment plans were designed on the DIBH image, using 6 MV, 8-field opposed-field intensity-modulated radiation therapy (IMRT) or dual - arc Volumetric Modulated Arc Therapy (VMAT). The hypofractionated dose schedule was 42.7 Gy/16Fx (PTV)+8 Gy/3Fx (GTV boost). DIBH images and structure sets were then exported to ETD.

#### 2.3.4 Positioning and position verification

**Figure 2:**
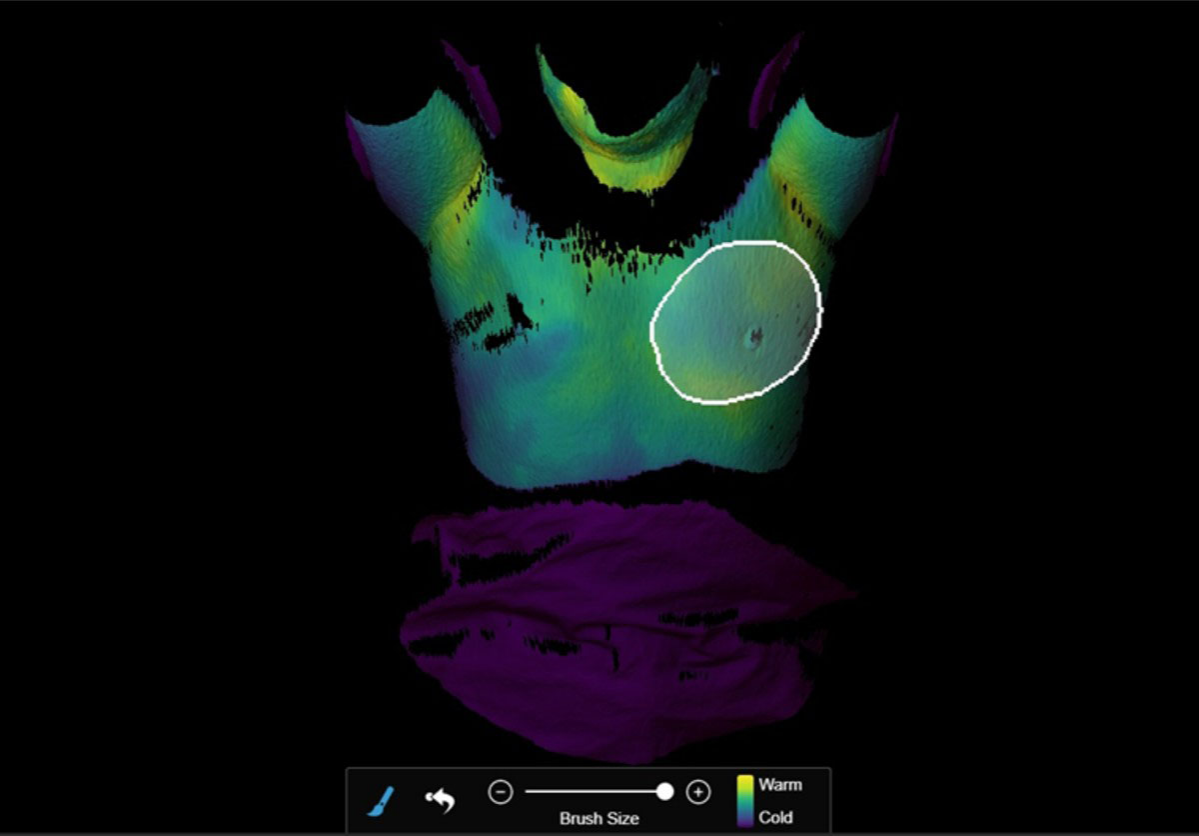
ROI on the affected breast surface for tracking.

During the first three radiotherapy fractions, patient setup was initially performed according to body surface cross-markers. The region of interest (ROI) was defined as the PTV surface area of the affected breast on thermo-optical surface images (**Figure 2**).

**Figure 3:**
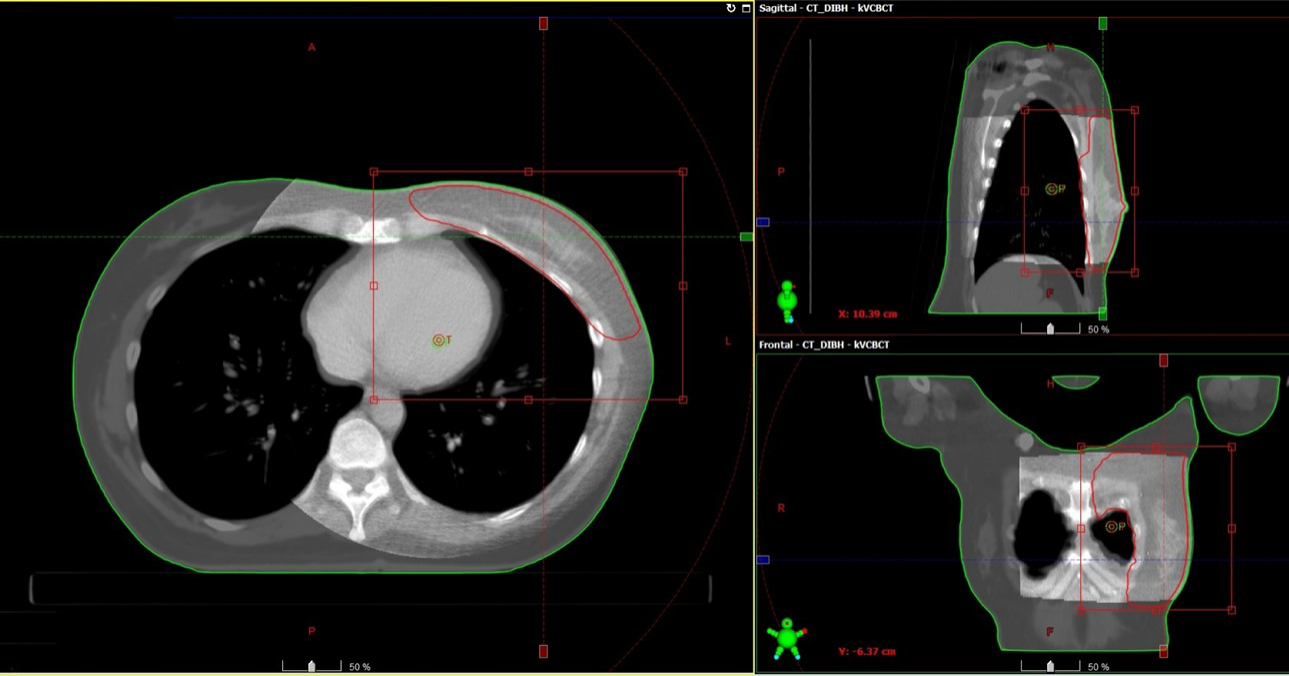
Align the CBCT with the positioning CT images.

Patients were instructed to perform deep inspiration to attain the DIBH scan reference position; inhalation amplitude was regulated via the therapist’s verbal commands and an in-room patient-facing visual screen. One set of X-ray plain images was acquired using ETD, which automatically registered with the digitally reconstructed radiograph (DRR) generated from the planning DIBH-CT dataset, with registration finished within 2 s. Following manual verification and confirmation, the treatment couch was adjusted for positional correction. After a 30-second rest interval, CBCT scanning was conducted under identical breath-hold conditions. The CBCT scan employed the Full Fan 200°fast-scan mode, completed within 35 s, with registration restricted to the affected breast and adjacent regions. The registration ROI was delineated as follows: medial border at half the sternum, lateral border encompassing the affected chest wall, cranial-caudal border extending 10 mm beyond the PTV, anterior border outside the chest wall, and posterior border at the mid-axillary line (**Figure 3**). Automatic registration combined with manual correction was applied to align the intra-treatment DIBH-CBCT images with the planning CT images, and the six-dimensional setup errors were recorded.

In subsequent treatment fractions, patients were positioned using body surface cross-markers under free-breathing conditions. Under the DIBH state with a tolerance of ±2 mm, ETD was utilized to verify the positional accuracy via X-ray imaging, followed by automated treatment couch correction for setup errors.

At the 6th, 9th, 12th, and 15th fractions, DIBH-CBCT images were acquired both before treatment and during treatment (upon completion of the 5th field), then registered to the planning CT images. The six-dimensional setup deviations were documented accordingly. Meanwhile, total treatment duration (from patient couch placement to beam completion) was recorded.

#### 2.3.5 In-treatment monitoring

After image correction, the thermo-optical surface monitoring system continuously acquired real-time surface images throughout treatment, enabling uninterrupted monitoring of intra-fraction patient displacement. Tolerance thresholds were set at ±2 mm for LAT, LNG, VRT translational errors and ±2°for pitch, roll, Rtn rotational errors. Beam delivery was immediately interrupted if surface motion exceeded these thresholds, and treatment resumed only after the patient’s breathing was re-adjusted to within the tolerance range. In the 8-field IMRT treatment, the 2nd, 5th, and 8th fields represent the early, middle, and late stages of the treatment, respectively. Therefore, the thermal optical surface monitoring deviation values were recorded at these three time points. After the 5th field, X-ray radiographs were taken simultaneously for position correction, and the six-dimensional bed displacement values were recorded to observe the mid-treatment drift and the correction effect. Translational deviations detected by thermo-optical surface monitoring were recorded three times each during the 2nd, 5th, and 8th IMRT fields. Prolonged supine positioning during radiotherapy often led to unavoidable patient body torsion and deformation. If such displacement or deformation surpassed the monitoring tolerance and could not be corrected by patient repositioning alone, an additional set of X-ray plain images was acquired after the 5th field. Automatic registration and couch correction were subsequently performed, and the corresponding six-dimensional couch displacement values were documented.

### 2.4 Evaluation indicators and analysis methods

Data processing and graphing were performed using SPSS 27.0 and Origin 2024. The primary evaluation indices and statistical analyses are detailed below.

#### 2.4.1 Primary evaluation indices

Inter-fraction residual setup error: Following X−ray image correction and couch adjustment, DIBH-CBCT was used as the gold standard. Couch shift deviations were derived via image registration to validate the correction accuracy of oblique X−ray guidance. Intra-fraction setup offset: Thermo−optical surface monitoring was employed to record patient displacement, thereby evaluating intra-fraction setup stability. Correlations between displacement and treatment duration were analyzed in conjunction with intra-fraction X−ray correction data. Individual and group error components: Based on CBCT and X−ray correction datasets, inter-fraction individual systematic errors, inter-fraction random errors, and intra-fraction random errors (six−dimensional) were calculated. Group−level errors were summarized to facilitate target volume margin calculation.

#### 2.4.2 Statistical analysis

Traditional parametric tests (e.g., t−test) are invalid for this dataset due to the nesting and intra−patient correlation inherent in repeated measurements from the same subject, which violates the independence assumption. Consequently, linear mixed models (LMM) were adopted as the primary analytical method, with patient ID included as a random effect to account for within−patient correlation. *P* < 0.05 was considered statistically significant. The specific steps included: Inter-fraction setup accuracy verification: The mean and standard deviation of CBCT residual errors were computed. LMM was applied to assess whether the mean error differed significantly from zero, as well as the impact of breast volume on setup accuracy. Intra-fraction setup stability evaluation: Thermo−optical monitoring and image correction data were analyzed. Treatment stability was evaluated using offset data across different treatment fields. LMM was utilized to examine the association between setup offset and treatment duration. PTV margin calculation: The van Herk formula was applied. Individual and group systematic errors and random errors were computed to determine the required PTV margins required for the study population.


$$\text{MPTV}=2.5*\Sigma +0.7*{\text{sqrt}(\text{σ}}_{\text{inter}}^{2}+{\text{σ}}_{\text{intra}}^{2})$$

## 3 Results

31 patients were enrolled, with 175 raw treatment datasets collected. After exclusion, 30 datasets were discarded due to incomplete data collection or excessive errors caused by excessive body motion, inadequate breath control and unstable breath-hold during treatment. Another 12 datasets were excluded owing to unsuccessful CBCT scanning, which resulted from an off-center or excessively low treatment couch after technician setup, X−ray imaging and calibration. Finally, valid datasets were obtained as follows: 144 inter−fraction setup errors derived from CBCT scans; 127 intra−fraction setup errors obtained via real−time thermo−optical monitoring + CBCT registration; and 135 intra−fraction setup error datasets from intra−fraction X−ray image correction.

### 3.1 Comparison results of inter-fraction CBCT setup errors

**Figure 4:**
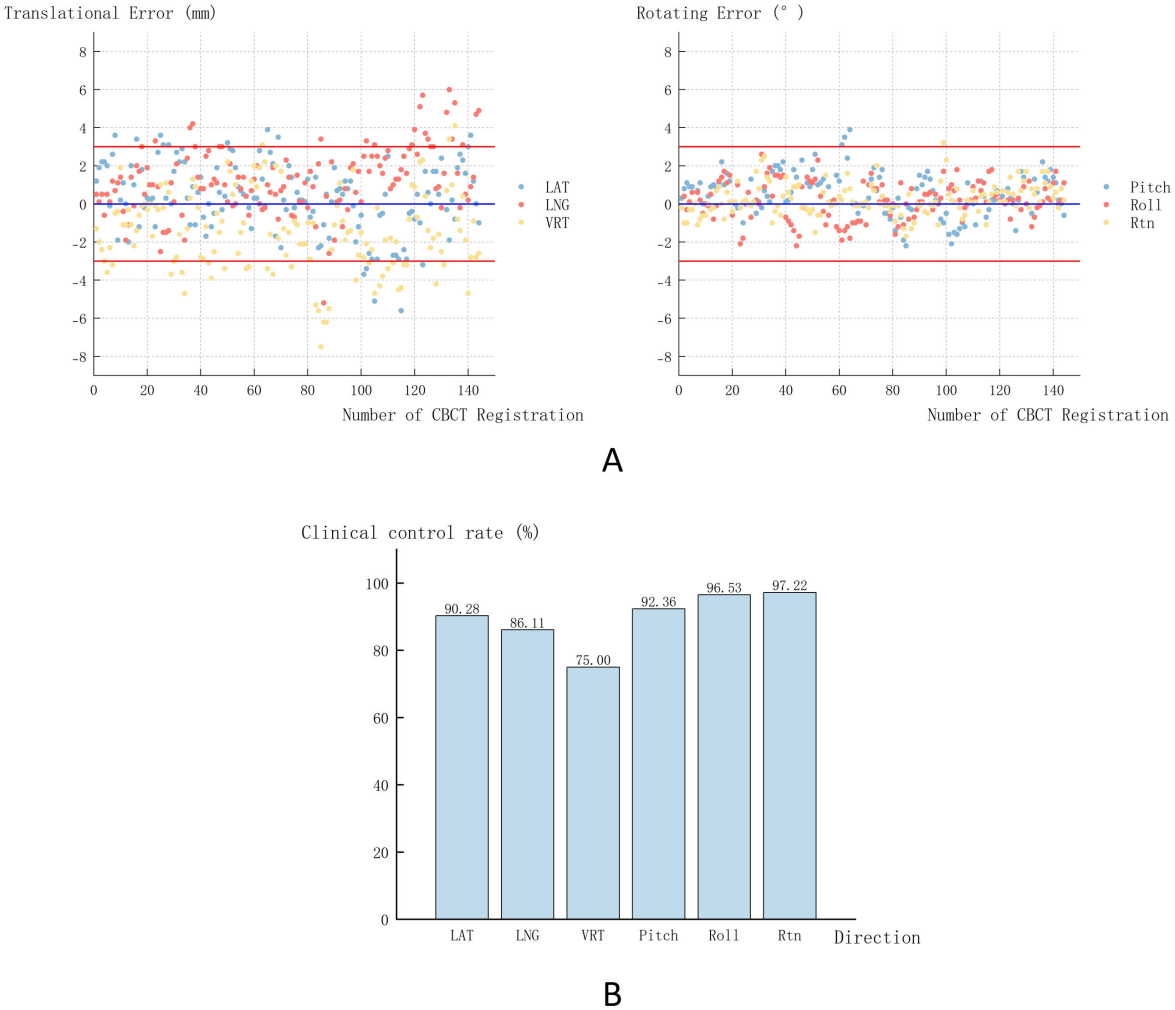
Inter-fractional distribution and clinical control rate of six-dimensional CBCT setup errors. **(A)** Distribution of translational and rotational setup errors. **(B)** Clinical control rates in each translational and rotational direction.

Inter−fraction CBCT setup deviation data comprised six−dimensional translational and rotational errors. The inter−fraction setup errors (Mean ± SD) were as follows: LAT: (0.3 ± 1.9) mm, LNG: (1.2 ± 1.7) mm, VRT: (-1.5 ± 2.1) mm; Pitch: (0.5 ± 1.1)°, Roll: (0.2 ± 1.0)°, Rtn: (0.2 ± 0.8)°. The proportions of patients with translational errors ≤ 3 mm in the LAT, LNG and VRT directions, and absolute rotational deviations ≤ 2° in the pitch, roll and Rtn directions were 90.28%, 86.11%, 75.00%, 92.36%, 96.53% and 97.22%, respectively (**Figure 4**). Following inter−fraction X−ray image correction, the six−dimensional mean setup errors showed no significant difference from zero (*P* > 0.05) (**Table 1**). Neither affected breast volume nor contralateral breast volume exerted a significant effect on setup accuracy (*P* > 0.05) (**Table 2**).

**Table 1:** LMM analysis of interfractional CBCT setup errors: est.: estimated value; SE: standard error.

*N* = 144		Fixed effects			Randomizing effect
Variate	Parameter	Intercept	Age	Treatment fractionation	Patient ID
LAT	Est.	0.608	-0.007	0.064	1.951
	SE	1.973	0.040	0.062	0.634
	t Value	0.308	-0.178	1.029	/
	*P* Value	0.760	0.860	0.306	0.002
LNG	Est.	3.258	-0.043	-0.106	1.248
	SE	1.672	0.034	0.064	0.447
	t Value	1.949	-1.265	-1.650	/
	*P* Value	0.061	0.216	0.102	0.005
VRT	Est.	-2.690	0.026	0.018	2.769
	SE	2.298	0.046	0.065	0.871
	t Value	-1.171	0.557	0.282	/
	*P* Value	0.252	0.582	0.779	0.001
Pitch	Est.	-0.410	0.018	0.101	0.768
	SE	1.192	0.024	0.031	0.241
	t Value	-0.344	0.746	3.282	/
	*P* Value	0.733	0.462	0.001	0.001
Roll	Est.	1.398	-0.017	0.061	0.272
	SE	0.800	0.016	0.033	0.102
	t Value	1.747	-1.075	1.854	/
	*P* Value	0.091	0.291	0.066	0.008
Rtn	Est.	0.138	-0.001	0.033	0.331
	SE	0.835	0.017	0.029	0.111
	t Value	0.165	-0.063	1.118	/
	*P* Value	0.870	0.951	0.266	0.003

**Table 2:** LMM analysis of breast volume (affected and contralateral sides) on interfractional image registration accuracy: F-value: the ratio of between-group variance to within-group variance, utilized to test the significance of fixed effects (in this study, “breast volume”); *P* value: employed to determine the statistical significance of the *F* value.

*N* = 144		Translation			Rotate		
		LAT	LNG	VRT	Pitch	Roll	Rtn
Affected Breast Volume	*F* Value	0.555	0.108	0.128	0.138	0.942	0.005
	*P* Value	0.463	0.745	0.724	0.714	0.342	0.944
Contralateral Breast Volume	*F* Value	0.392	0.205	0.854	0.099	2.339	0.858
	*P* Value	0.537	0.655	0.365	0.756	0.141	0.363

### 3.2 Comparative analysis of intra-fraction patient positioning stability

**Figure 5:**
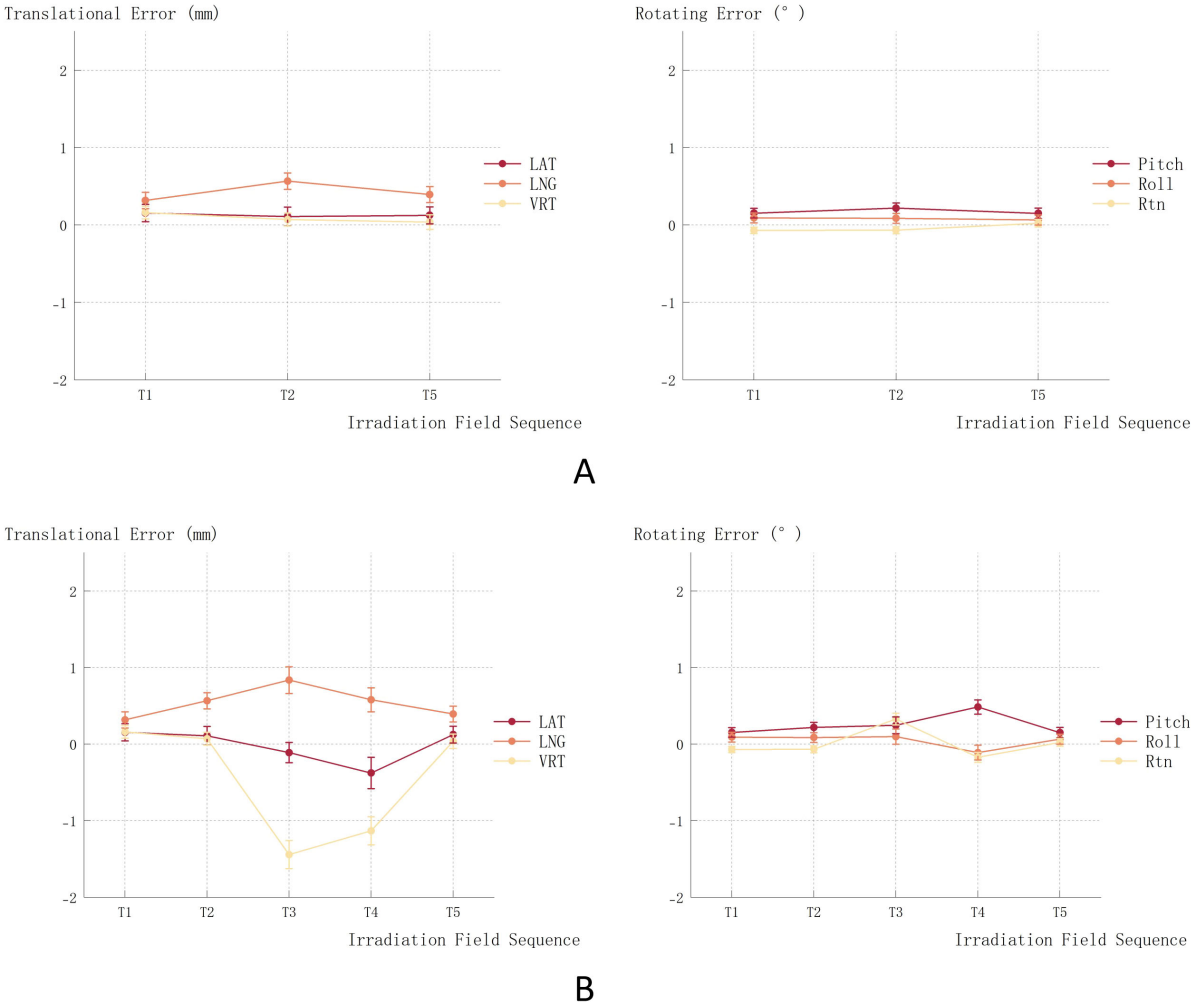
Intra-fraction patient displacement monitoring. **(A)** Thermo-optical surface monitoring errors at different treatment fields. **(B)** Changes in setup errors before and after mid-treatment X-ray image-guided correction.

No statistically significant differences were observed in six−dimensional setup errors across different treatment durations (*P* > 0.05; **Table 3**). During treatment, thermo-optical surface monitoring offsets were recorded at three time points: the 2nd, 5th, and 8th treatment fields. The 2nd field represents the early stage of treatment, the 5th field represents the middle stage, and the 8th field represents the late stage. A DIBH-CBCT scan was performed at the mid-treatment point (after the 5th field), followed by one session of X-ray image-guided correction, with the corresponding couch shift values documented. Statistical analysis was performed on five sequences (T1–T5), corresponding to thermo-optical errors at the 2nd and 5th fields, post-5th-field CBCT errors, X-ray correction errors, and thermo-optical errors at the 8th field (**Table 4**). The line chart (**Figure 5**) reveals that all thermo-optical monitoring errors were within clinical tolerance. At the mid-treatment point (5th field), the errors in sequence T2 were higher than those in T1 (**Figure 5A**). Following X-ray image-guided correction, the errors in sequence T5 decreased compared with the preceding measurements (**Figure 5B**).

**Table 3:** Linear mixed model analysis of intra-fractional X-ray imaging correction positioning errors with respect to treatment time: F-value: the ratio of between-group variance to within-group variance, utilized to test the significance of fixed effects (in this study, “ treatment time “); *P* value: employed to determine the statistical significance of the *F* value.

*N* = 135		Translation			Rotate		
		LAT	LNG	VRT	Pitch	Roll	Rtn
Treatment Time	*F* Value	1.370	0.748	3.671	0.126	0.002	0.718
	*P* Value	0.244	0.389	0.058	0.723	0.964	0.398

**Table 4:** Comparative analysis of intra-fraction patient setup and registration accuracy.

*N* =127	T1	T2	T3	T4	T5
LAT(mm)	0.15 ± 1.26	0.11 ± 1.37	-0.11 ± 1.49	-0.38 ± 2.26	0.12 ± 1.24
LNG(mm)	0.32 ± 1.19	0.57 ± 1.19	0.84 ± 1.97	0.58 ± 1.73	0.39 ± 1.16
VRT(mm)	0.16 ± 0.92	0.07 ± 0.95	-1.44 ± 2.06	-1.13 ± 2.01	0.04 ± 1.06
Pitch(°)	0.15 ± 0.71	0.22 ± 0.76	0.25 ± 1.23	0.49 ± 1.02	0.15 ± 0.78
Roll(°)	0.09 ± 0.73	0.09 ± 0.72	0.10 ± 1.13	-0.11 ± 1.06	0.07 ± 0.73
Rtn(°)	-0.07 ± 0.47	-0.07 ± 0.53	0.33 ± 0.86	-0.17 ± 0.72	0.02 ± 0.56

### 3.3 Dosimetric verification of imaging modalities

A total of 271 CBCT scans were acquired in this study (144 inter-fraction, 127 intra-fraction). Scanning parameters were tailored to patient body habitus: tube voltage 125 kV, tube current-time product 270–495mAs. The corresponding CBCT effective dose ranged from 3.98 to 7.46 mSv, with a mean of 6.93 mSv. Additionally, 279 planar X-ray image corrections were performed (144 inter-fraction, 135 intra-fraction). X-ray exposure parameters were 100–120 kV and 8–20mAs. The effective dose per set of planar X-ray images was 9.4–36.5μSv, with a mean of 32.8μSv.

The effective dose (ED) of CBCT is calculated based on the computed tomography dose index (CTDI) provided by the accelerator and the scan length. The X-ray flat-panel dose is based on the effective dose value provided by the ETD integrated guidance system. The calculation formula for the ED of CBCT:


ED (mSv)=CTDI vol (mGy)×Scan Length (cm)×Conversion Factor (k)

Since the conversion factor (k) is related to the examination part. Different parts of the same individual have different sensitivities to the same radiation dose, so the value of k also varies. This study refers to the idea in AAPM TG-75 imaging-guided radiotherapy dose management regarding the estimation of effective dose using CT-type images multiplied by the site conversion coefficient (16), and combines the commonly used effective dose conversion coefficient for adult chest CT. The value of k is set as 0.014 mSv·mGy-1·cm-1. If a multi-center comparison is to be conducted later, the measured dose parameters of this model or the medical physics quality control records should be used for correction as a priority.

### 3.4 PTV margin expansion

According to the van Herk formula, the calculated PTV margins (**Table 5**) were 5.10 mm (LAT), 5.22 mm (LNG), and 5.78 mm (VRT).

**Table 5:** Calculation of PTV margin expansion: Σ (population systematic error): the standard deviation of the patient-specific systematic errors (mean deviation of errors in the same dimension across all treatment fractions).

n=127	Translation		
	LAT	LNG	VRT
Σ	1.48	1.53	1.75
σ_inter_	1.15	1.31	1.19
σ_intra_	1.65	1.53	1.63
MPTV	5.10	5.22	5.78

σ_inter_ (inter-fraction random error): the mean of patient-specific random errors (standard deviation of errors in the same dimension across all treatment fractions). σ_intra_ (intra-fraction random error): the mean of patient-specific intra-fraction random errors (standard deviation of same-dimension errors corrected by CBCT or X-ray imaging within all treatment fractions).

## 4 Discussion

Achieving optimal precision in radiotherapy hinges on guaranteeing the accuracy and reproducibility of patient setup. Image-Guided Radiation Therapy (IGRT) and Surface Guided Radiation Therapy (SGRT) are now widely adopted in clinical practice, serving as pivotal modalities to reduce setup errors and elevate treatment precision. In breast cancer radiotherapy, the combination of DIBH and OSMS is routinely implemented to manage respiratory motion and ensure accurate treatment delivery. Building upon this foundation, this research utilized CBCT as the gold standard to investigate the feasibility of applying ETD in DIBH radiotherapy for patients after breast-conserving surgery. Surface guidance itself is not a new concept in breast cancer DIBH radiotherapy; therefore, the clinical relevance of the present study should not be interpreted as the use of surface monitoring alone. Instead, the value of ETD lies in its integrated workflow, which combines thermo-optical surface tracking with stereoscopic X-ray guidance (15). Conventional surface-guidance systems typically employ 2–3 camera projection of structured light, which often leads to the obstruction of the structured light beam by the gantry at certain specific treatment angles. They mainly provide continuous external alternative monitoring. In contrast, ETD combines optics with thermal imaging for complementation. It can replace multiple cameras with a single camera to achieve unobstructed and stable tracking at any gantry angle, and allows real-time non-ionizing surface monitoring, automatic beam interruption when predefined thresholds are exceeded, and intermittent image-based six-dimensional correction when additional anatomical verification is required. This combined workflow is particularly relevant for breast DIBH radiotherapy, because a reproducible external surface position may not fully represent internal anatomical alignment. Therefore, the present study aimed to evaluate ETD as an integrated monitoring-and-correction strategy rather than as a replacement for all existing surface-guidance techniques.

This study analyzed the correlation between ETD positioning, monitoring data and the CBCT gold standard (**Figure 4**). The results illustrated that after X-ray image correction, more than 90% of residual setup errors were controlled within 3 mm for translational direction (LAT) and 2°for all rotational directions (Pitch, Roll, Rtn). Further LNM analysis (**Table 1**) demonstrated that the mean residual errors across all six dimensions were close to zero following X-ray correction. This suggests that the X-ray system offers calibration accuracy comparable to CBCT, supporting its utility as a feasible alternative to CBCT for pretreatment setup verification in breast cancer DIBH radiotherapy. Moreover, no significant correlation was found between bilateral breast volume and inter-fraction setup accuracy across six dimensions (*P* > 0.05) (**Table 2**), indicating that breast volume has no impact on inter-fraction setup precision.

ETD was utilized for real-time monitoring and calibration of intra-fraction setup stability. Results presented in **Table 3** demonstrated no significant linear positional drift in patients throughout treatment fractions. Notably, the vertical (VRT) direction yielded a P-value of 0.058; although this did not reach the conventional statistical significance threshold (*P* < 0.05), it indicated a potential trend of positional drift over time. This finding is partially inconsistent with those reported by Wiant and Jianfeng Liu et al. (17, 18) who observed that prolonged treatment duration was associated with increased intra-fraction patient motion, which was most prominent in LNG, followed by VRT. The discrepancy can be attributed to the immobilization strategy adopted in this study: a breast board combined with hip and foot supports was employed to stabilize the patient’s torso and lower extremities, thereby restricting displacement in the LNG and VRT directions. Additionally, the thermo-optical surface monitoring system was set with a stringent tolerance threshold. Beam delivery was immediately interrupted once deviations exceeded the threshold and only resumed after re-calibration, which effectively minimized intra-fraction patient motion. Analysis of intra-fraction optical monitoring data (**Figure 5A**) further substantiates the above conclusion, confirming that ETD enables real-time, continuous and effective monitoring of surface displacement during DIBH radiotherapy for breast cancer. Therefore, the monitoring accuracy of ETD satisfies the clinical requirements of DIBH radiotherapy, keeping patient motion within clinically acceptable tolerance ranges. Furthermore, supplementary X-ray image correction performed in the latter phase of each treatment fraction can further reduce setup deviations and improve treatment precision (**Figure 5B**).

Although CBCT is widely adopted as the gold standard for image-guided radiotherapy, it is limited by prolonged scanning durations and relatively high radiation exposure. Relying exclusively on inter-fraction CBCT may fail to detect positional drift that occurs in the later phase of a treatment fraction (**Figure 5B**). In this study, the mean effective dose per CBCT scan was 6.93 mSv, while the average dose per set of planar X-ray images was merely 32.8μSv. For example, performing two sets of X-ray verification per DIBH treatment fraction yields a dose of 65.6μSv. Over an entire 19-fraction treatment course, the cumulative radiation dose amounts to approximately 1.25 mSv, accounting for only about 20% of the dose from a single CBCT scan. This is consistent with the findings reported by Murphy et al. (16) regarding dose management in image-guided radiotherapy, which documented that the effective dose of a single CBCT examination ranges from 3 to 8 mSv. Accordingly, it is critical to account for the cumulative radiation dose from serial CBCT scans and prudently restrict the scan frequency throughout treatment to mitigate potential long-term radiation risks. In contrast, ETD enables pre-treatment and intra-treatment image verification with markedly lower radiation exposure, while sustaining robust and reliable positioning accuracy. The role of ETD should also be interpreted in the context of modern breast radiotherapy techniques. Recent planning strategies, such as RapidArc Dynamic, mainly aim to improve the planned dose distribution by integrating dynamic collimator rotation and static angle ports during VMAT delivery. Yuen et al. reported that RapidArc Dynamic reduced contralateral breast mean dose by 27.5%, left-lung mean dose by 13.0%, and mean heart dose by 21.6% compared with conventional RapidArc in hypofractionated whole-breast irradiation for Chinese female patients (19). These results suggest that advanced planning techniques can improve organ-at-risk sparing at the planning stage. In contrast, ETD does not directly optimize the treatment plan or improve dose conformity during planning. Its potential benefit lies in improving the reproducibility and accuracy of treatment delivery under DIBH conditions through continuous surface monitoring and intermittent X-ray correction. Therefore, ETD and advanced planning techniques should be regarded as complementary: optimized planning may reduce the intended dose to organs at risk, whereas reliable surface/image guidance may help ensure that this dosimetric advantage is maintained during actual treatment.

Patient setup errors directly determine the formulation of PTV margins, with a 5 mm margin commonly adopted in clinical practice. In this study, the van Herk margin formula MPTV = 2.5***Σ* **+ 0.7***σ*** was utilized to derive the optimal PTV margins for breast cancer DIBH radiotherapy with ETD. Analysis of the data demonstrated that the recommended margins were 5.1 mm (LAT), 5.2 mm (LNG), and 5.8 mm (VRT) for the three translational directions (**Table 5**). These findings are consistent with those reported by Volker et al. (20) who analyzed 303 fractions from 23 left-sided breast cancer patients treated with DIBH plus SGRT. By applying the van Herk formula to account for both inter-fraction and intra-fraction setup errors, they recommended PTV margins of 4 mm, 6 mm, and 4 mm in the LAT, LNG, and VRT respectively.

This study has several limitations. First, the sample size was relatively modest, and the present conclusions require further validation in large-scale cohort studies. Second, the setup procedure was susceptible to variability stemming from operator technique, patient respiratory patterns, and equipment performance. Accordingly, it is essential to standardize imaging verification protocols and optimize patient respiratory training. Third, this research was restricted to patients who underwent breast-conserving surgery followed by DIBH radiotherapy. The generalizability of the findings to patients treated with mastectomy remains to be explored. Last but not least, given that the breast is a superficial anatomical site, ETD achieved satisfactory real-time monitoring performance. Nevertheless, further investigation is required to determine whether these results can be extended to deepest targets, such as pulmonary and hepatic lesions. Third, the present study focused on inter-fraction setup correction and intra-fraction motion monitoring but did not directly reconstruct or verify the delivered dose distribution. Positional accuracy does not necessarily guarantee dosimetric accuracy. A recent study on biological *in vivo* three-dimensional dose distribution verification for lung cancer demonstrated that respiratory pattern inconsistency can substantially affect dose delivery: the gamma passing rate under CT-simulation breathing was approximately 93.61%, whereas irregular patterns such as Cheyne-Stokes breathing, yawning, and coughing reduced the passing rates to 87.71%, 81.35%, and 75.31%, respectively (21). Although breast DIBH radiotherapy differs from free-breathing lung radiotherapy, variations in breath-hold level, chest wall deformation, and imperfect correspondence between external surface surrogates and internal anatomy may still affect the delivered dose. Therefore, the findings of this study should be interpreted as evidence of improved positional monitoring and correction rather than direct evidence of delivered-dose accuracy. Future studies should integrate ETD-derived surface motion and X-ray correction data with EPID-based dose reconstruction, *in vivo* dosimetry, or adaptive dose accumulation to determine whether improved positional control can be translated into improved delivered-dose accuracy.

In conclusion, DIBH+ETD demonstrates considerable clinical potential for radiotherapy in patients with breast cancer following breast-conserving surgery. It achieves calibration accuracy comparable to CBCT, while conferring a markedly lower radiation dose, thus representing a feasible alternative for inter-fraction setup verification. Additionally, this system enables stable and effective intra-fraction motion monitoring and supports mid-treatment repositioning to further improve treatment accuracy. Moreover, the proposed strategy may yield technical benefits in other clinical scenarios, including radiotherapy for head and neck tumors with minimal positional variation, as well as pulmonary and hepatic malignant tumors which are vulnerable to being affected by respiratory motion. Nevertheless, broader clinical implementation and application require further in-depth investigation and prospective validation.

## Data Availability

The raw data supporting the conclusions of this article will be made available by the authors, without undue reservation.
